# Supplementary material for: Epidermal threads reveal the origin of hagfish slime
Source: eLife. 2023 Mar 10;12:e81405. doi: 10.7554/eLife.81405 (PMC10005773; doi:10.7554/eLife.81405)
Supplement: Supplementary file 1. — (A) Morphological parameters of slime glands of Pacific Hagfish. (B) Comparison of fibrosity between defensive slime and epidermal slime. [file elife-81405-supp1.docx]

**Supplementary File 1**

**A. Morphological parameters of slime glands of Pacific Hagfish.** Values correspond with one full exhaustion of a single gland. †, mean values of morphological variables of full and newly emptied slime glands from literature (Schorno et al., 2018). ‡, single GTC thread length 18 cm was assumed (see Zeng et al., 2021).

|  | **Full gland** | **Newly emptied gland** | **Ejected exudate** |
| --- | --- | --- | --- |
| Minor axis, Φ_a_ (mm) † | 2.62 | 1.77 | / |
| Major axis, Φ_b_ (mm) † | 3.77 | 2.55 | / |
| Volume (mm^3^) | 13.6 | 4.2 | 9.4 |
| GTC area density, σ (mm^-2^) | 126.5 | 98.8 | / |
| Total GTC number, N_GTC_ | 19300 | 4100 | 15200 |
| Total thread length (m) ‡ | 3474 | 738 | 2736 |
| GMC area proportion, $\delta$ (%) † | 56.6 | 83.5 | / |
| GMC volume (mm^3^) | 7.7 | 3.5 | 4.2 |

**B. Comparison of fibrosity between defensive slime and epidermal slime.** Values for defensive slime correspond with one full exhaustion of a single gland. Values for epidermal slime correspond with 1 mm^2^ of skin surface, with volume approximated based on the thickness of epidermis and experimentally measured relative water content (see main text).

|  | Ejected exudate per gland | Fully-deployed slime per gland | Epidermal slime per 1 mm^2^ skin |
| --- | --- | --- | --- |
| Total thread length (mm) | 2736000 | 2736000 | 955 |
| Volume (mm^3^) | 9.4 | 234000 | 0.12 |
| Fibrosity index (mm/mm^3^) | 651429 | 12 | 8024 |
